# Targeting Inflammation and Immunosenescence to Improve Vaccine Responses in the Elderly

**DOI:** 10.3389/fimmu.2020.583019

**Published:** 2020-10-14

**Authors:** Branca Pereira, Xiao-Ning Xu, Arne N. Akbar

**Affiliations:** ^1^HIV/GUM Directorate, Chelsea and Westminster Hospital NHS Foundation Trust, London, United Kingdom; ^2^Faculty of Medicine, Imperial College London, London, United Kingdom; ^3^Division of Medicine, University College London, London, United Kingdom

**Keywords:** aging, immunosenescence and inflammaging, vaccine, T lymphocytes, anti-inflammatories

## Abstract

One of the most appreciated consequences of immunosenescence is an impaired response to vaccines with advanced age. While most studies report impaired antibody responses in older adults as a correlate of vaccine efficacy, it is now widely appreciated that this may fail to identify important changes occurring in the immune system with age that may affect vaccine efficacy. The impact of immunosenescence on vaccination goes beyond the defects on antibody responses as T cell-mediated responses are reshaped during aging and certainly affect vaccination. Likewise, age-related changes in the innate immune system may have important consequences on antigen presentation and priming of adaptive immune responses. Importantly, a low-level chronic inflammatory status known as inflammaging has been shown to inhibit immune responses to vaccination and pharmacological strategies aiming at blocking baseline inflammation can be potentially used to boost vaccine responses. Yet current strategies aiming at improving immunogenicity in the elderly have mainly focused on the use of adjuvants to promote local inflammation. More research is needed to understand the role of inflammation in vaccine responses and to reconcile these seemingly paradoxical observations. Alternative approaches to improve vaccine responses in the elderly include the use of higher vaccine doses or alternative routes of vaccination showing only limited benefits. This review will explore novel targets and potential new strategies for enhancing vaccine responses in older adults, including the use of anti-inflammatory drugs and immunomodulators.

## Introduction

Human aging is associated with a general decline in physiological functions and increased susceptibility to disease. A dysregulation of the immune system, known as immunosenescence, is characteristic of aging and has been linked with negative clinical outcomes in older adults (1). One of the most appreciated consequences of immunosenescence is an impaired response to new infections and vaccination in older people (2). Four vaccines are currently recommended for individuals over 65 years of age to protect against infections that disproportionately affect older adults, including influenza, herpes zoster, pneumococcal disease and tetanus and diphtheria. However, responses to these vaccines are often impaired in older individuals placing them at further risk of disease (3, 4). This has considerable implications for vaccination against emerging infectious diseases such as COVID-19 that have a disproportionately larger effect on older subjects (5).

While most studies report antibody responses as a correlate of vaccine efficacy, it is now widely appreciated that this may fail to identify important changes occurring in the immune system with age that may affect vaccine efficacy (6, 7). The impact of immunosenescence on vaccination goes beyond the defects on T and B cell responses and changes in innate immunity and increased systemic inflammation, also referred to as inflammaging, may have additional consequences on vaccine efficacy (8). While the mechanisms of immune aging are not yet fully understood, it is now apparent that this process is dynamic and multifaceted, with a decline in many primordial functions but also gain of new functions as well as changes in the microenvironment. Globally, age-related changes in the immune system are better described as a remodeling than a decline in immune functions (9). A better understanding of the full spectrum of changes characterizing immunesenescence is fundamental to the development of novel and improved vaccines for older adults.

## How Can Immunosenescence and Inflammation Affect Vaccine Responses?

Changes affecting both innate and adaptive immune function with age may lead to impaired vaccine responses in older people. Immunosenescence is primarily linked to the involution of primary lymphoid organs (bone marrow and thymus), resulting in depletion of the peripheral pool of naive B and T cells (10). To maintain peripheral cell numbers, there is a clonal expansion of antigen-experienced cells resulting in extreme differentiation and altered functionality (11). Consequently the immune space becomes filled with antigen-specific memory cells leading to a contraction of the immune repertoire and impaired responses to neo-antigens (12). In parallel with this, the effects of aging on hematopoiesis result in a lineage skewing towards an increase in myeloid versus lymphoid precursor (13). Although the numbers of most circulating innate immune cells may not be significantly reduced with age, alterations in their functionality have a particular impact on antigen presentation due to decreased antigen uptake, reduced phagocyte functions and altered cytokine production (13, 14). In addition to cell-intrinsic changes, alterations in the microenvironment including a low-grade chronic inflammatory status and architectural changes occurring in lymph nodes may play previously underappreciated roles in shaping vaccine responses with age (1, 15). Excessive baseline inflammation has been recently associated with poor responses to vaccination (16) however more research is needed to reconcile this evidence with the current paradigm that adjuvants enhance immune responses to vaccines by promoting local inflammation. It is plausible that stronger local inflammatory signals are needed to overcome background inflammation or that specific inflammatory pathways should be triggered to overcome local inhibitory responses. Thus a better understanding of the role of inflammation in vaccination and of the mechanisms of action of adjuvants is needed to be able to fine tune immune responses and selectively stimulate pathways that lead to long-lasting immune protection. In this review, we will describe the most recent data on the effects of aging on immune responses to vaccination and discuss, in light of the current knowledge, how can immunesenescence and inflammaging be targeted to improve vaccine responses in older adults.

### Age-Related Changes in Adaptive Immunity

#### Changes in the T Cell Compartment

The effects of aging are particularly evident in the T cell compartment and reduced vaccine responses in older people are, at least in part, due to defective T cell memory responses with age (17). Different mechanisms may be contributing to reduced T cell responsiveness with age (18), but the loss of proliferative capacity (19) and decreased TCR function (20–22) and TCR diversity (23) are certainly determining factors. Prior antigen exposure, in particular latent viral infections such as cytomegalovirus (CMV) and Epstein-Barr Virus (EBV) have a significant impact on immunosenescence by shaping the immune repertoire with large proportions of terminally differentiated cells with reduced proliferative capacity and features of replicative senescence (24–26). Despite this, data on the impact of CMV infection on vaccine responses are controversial, with studies showing an association between CMV-seropositivity and impaired antibody responses to vaccination in older adults (3, 27) while others have found enhanced antibody responses to influenza vaccination in CMV-seropositive compared to CMV-negative individuals (28, 29). Nevertheless, it has been shown that CMV seropositivity is a better predictor of a decline in T cell responses to influenza challenge rather than antibody responses in vaccinated older adults (30, 31). When using functional assays of CD8^+^ T cell cytolytic activity upon *ex vivo* influenza challenge, CMV-seropositivity was associated with impaired cytolitic responses to influenza, measured by granzyme B levels in virus-challenged T cells (30, 31).

Mechanistically, we have described that highly differentiated T cells with features of senescence exhibit decreased TCR responsiveness as a results of loss of key components of the TCR signalossome (20, 22). Interestingly, these cells concomitantly express NK lineage receptors and acquire TCR-independent functionality (32). Thus, non-proliferative senescent-like T cells, in particular CD8^+^ T cells, are reprogrammed to acquire broad, innate-like killing activity regulated by a group of stress sensing molecules known as sestrins (32). Studies in human centenarians have found an expansion of these NK-expressing T cells in old individuals compared to young (33) while others have shown that the expression of NK cell markers on CD8^+^ T cells is particularly evident in individuals with high levels of CD57, indicative of an aged immune system (34). The biological significance of the acquisition of innate-like receptors and functions by T cells is unclear, but we believe that this may serve as a beneficial adaptation to ensure broad and rapid effector function with age, independently of antigen-specificity, and this may represent a relatively unexplored opportunity to enhance vaccine-elicited immunity (35, 36). Despite the loss of proliferative potential, aged T cells are metabolically active and exhibit increased production of pro-inflammatory cytokines and thus may have detrimental effects on the tissue microenvironment, contributing to age-associated low-grade inflammation (37–39).

#### Changes in the B Cell Compartment

As with T cells, there is an age-dependent accumulation of late-stage memory B cells, while the circulating pool of naïve B cells progressively decreases, skewing the B cell repertoire and limiting the number of clones available to respond to novel antigens (40). B cells experience significant functional changes with age with reduced proliferative potential and impaired capacity for differentiation into plasma cells after antigen challenge (41). Senescent B cells have also been shown to spontaneously secrete pro-inflammatory cytokines contributing to age-related chronic inflammation and further immune dysregulation (42). Overall, these changes have been associated with poor health outcomes (43) and diminished responses to vaccination in old age (44). Several studies have shown that older adults have lower antibody responses following vaccination compared to younger adults and have been reviewed elsewhere (45). The quality of these antibody responses is also compromised with reduced diversity in the antibody repertoire (46, 47). This is particularly well described for influenza vaccination (48, 49), although responses to pneumococcal vaccines are equally compromised (50). Intrinsic defects of B cells, such as reduced somatic hypermutation and isotype switch as well as reduced numbers of plasma cells contribute to reduced antibody responses after vaccination and this correlates with decreased vaccine efficacy (41).

#### Changes in Innate Immunity With Age

Alterations in the phenotype and function of innate immune cells with age are increasingly well recognized (13, 14) and particularly relevant for vaccine-induced immune responses. Reduced chemotaxis, alterations in signaling pathways following antigen recognition and aberrant cytokine production have been described in neutrophils (51, 52), monocytes/macrophages (53, 54) and dendritic cells (DCs) (55, 56) derived from older persons further affecting their capacity to process and present antigen to T cells. Toll-like receptor (TLR) signaling has a crucial role in vaccination by linking innate and adaptive immune responses (57). Although the surface expression of TLRs does not show a consistent change with age, altered cytokine production and impaired downstream TLR signaling have been described in older adults (58). Interestingly, an age-dependent decrease in TLR function in human DCs has been linked with poor antibody responses to influenza immunization, providing evidence for the impact of an aging innate immune system in vaccine responses (59). Moreover, intracellular cytokine production in the absence of TLR ligand stimulation was elevated in cells from older compared with young individuals (59), suggesting a dysregulation of cytokine production that may contribute to age-related inflammation. Changes affecting the local microenvironment at the site of injection may have a significant effect on vaccine responses. Neutrophils and tissue-resident macrophages contribute to a pro-inflammatory environment at the site of vaccine injection that is important for recruiting other immune cells and for the priming of adaptive immune responses (60). However, as it will be discussed in more detail there is a growing appreciation that excessive local inflammation may be detrimental to vaccine responses (16).

The effects of age on the phenotype and function of NK cells have been described elsewhere (13, 61) and may as well affect the efficacy of vaccination in older people. As discussed later, NK cells have a previously unrecognized role in vaccination, contributing for protection during the early phases post-vaccination by mechanisms that involve the generation of innate immune memory (62). Thus, the effects of aging on cytotoxicity and cytokine secretion mediated by NK cells may have wider implications for immune responses to vaccination in older adults (63).

Age-related changes in innate T cells are less well described however a decreased frequency and change in phenotype of peripheral γδ T cells (64) and mucosal-associated invariant T (MAIT) cells (65) have been reported in older adults compared to young. Recently it has been described that MAIT cells in older adults have an increased baseline inflammatory profile that was associated with reduced *Escherichia coli*–specific responses in aged MAIT cells compared with their young adult counterparts (66).

### Inflammaging

Aging is associated with a chronic and systemic sterile inflammatory state termed inflammaging (67). This is supported by the findings of higher levels of tumor necrosis factor (TNF), IL-6 and other pro-inflammatory cytokines in the serum of older individuals compared to young (68, 69). A variety of stimuli may sustain inflammaging, not only chronic antigen stimulation by pathogens, but also activation of the inflammasome by endogenous cell debris and misplaced self-molecules and microbial translocation due to increased gut permeability (70). Although the innate immune system, in particular the monocyte-macrophage network are thought to be at the center of inflammaging (70, 71), accumulating evidence indicates that senescent cells in general, including senescent T and B cells have an important contribution with their senescent-associated secretory phenotype (SASP) (72). Regardless of the origin, this low-grade systemic inflammation is predictive of frailty and earlier mortality (73) and is an established risk factor for many age-related diseases including heart disease, age-related macular degeneration, type II diabetes, osteoporosis and cancer (74, 75).

There is accumulating evidence that increased chronic background levels of inflammation might be detrimental for vaccine responses (76–81). Nakaya et al. investigated gene signatures predictive of influenza vaccine responses in young and old adults and found that pre-vaccination signatures associated with T and B‐cell function were positively correlated with antibody responses at day 28 after vaccination, while monocyte‐ and inflammation‐related genes were negatively correlated with antibody responses (76). Similarly, studies on HBV vaccination in the elderly revealed that a more pronounced inflammatory gene expression profile at baseline predicted a poorer response to vaccination (77, 78). Our group has shown that older individuals exhibit reduced cutaneous immunity to varicella zoster virus recall antigen challenge associated with increased baseline local inflammation (79). Subsequently we demonstrated that infiltrating monocytes play a crucial role in the inhibition of cutaneous immunity, by a mechanism driven by increased cyclooxygenase 2 (COX2) expression and production of prostaglandin E2 (PGE2), ultimately leading to reduced proliferation of skin resident-memory T cells and reduced responses to antigenic stimulation (82). Overall, these findings support the concept that elevated baseline inflammation may have a significant role in the age-related hypo-responsiveness to vaccination and thus reducing background inflammation might be a promising strategy to enhance vaccine responses (83). This may be a particularly important consideration for older subjects who develop severe inflammation after SARS-Cov-2 where reducing inflammation may boost vaccine efficacy (84).

## Current Strategies to Improve Vaccine Effectiveness

Current recommendations for vaccination in older adults include vaccines against influenza, herpes zoster, pneumococcal disease and a booster against tetanus and diphtheria. Despite being able to mitigate the severity of the disease to some degree, these vaccines often fail to induce protective immunity in the elderly. Several approaches are currently in place to improve vaccine effectiveness in this population [discussed in detail elsewhere (4)] and largely focus on the use of adjuvants, higher antigen doses and alternative routes of immunization.

### Influenza Vaccines

Adjuvanted influenza vaccines are now the first choice for those over 65 years in countries such as Austria and the United Kingdom (UK) to overcome the low effectiveness of standard vaccines in the elderly (85). Data from the 2018/19 influenza season in the UK, the first season after the introduction of adjuvanted vaccines for persons above 65 years, demonstrated better protection from pneumonia-associated hospitalizations and laboratory-confirmed influenza cases with adjuvanted compared to non-adjuvanted vaccines (86). Studies have demonstrated that the addition of MF59® to influenza vaccine enhanced antibody production with increased seroconversion and seroprotection rates (87), improved antibody binding affinity and a more diverse antibody epitope repertoire (88) and induced broader serological protection against drifted strains (89) providing support for the use of adjuvants in influenza vaccination of older populations. Despite this, a study comparing cell-mediated immune responses to vaccination in adults ≥ 65 years old randomized to receive one of 4 seasonal influenza vaccines—standard subunit, MF59 adjuvanted subunit and split-virus vaccines given intramuscularly or intradermally—found no benefit of the MF-59 adjuvanted formulation over non-adjuvanted formulations delivered by intramuscular and intradermal routes (90).

Alternatively, the use of high-dose influenza vaccines in individuals over 65 years has also been shown to induce higher antibody titers and seroprotection rates compared to standard-dose vaccine (91), leading to their approval for clinical use in person aged 65 and older (92). Meta-analysis of randomized controlled trials (RCTs) showed that high-dose vaccines (split-virus and subunit recombinant hemagglutinin formulations) were more effective than standard-dose vaccines in preventing influenza-like illness, influenza hospitalization and all-cause mortality in adults ≥65 years old (93). When looking at T cell-mediated immune responses, high-dose influenza vaccines had little impact on the development of functional T cell memory in older adults compared to standard-dose vaccines (31).

Another approach to improve influenza vaccine immunogenicity in older people is the use of alternative routes of vaccination. Most vaccines are delivered by intramuscular or subcutaneous injection, bypassing the mucosal immune compartment. Intranasal and intradermal routes for influenza vaccination have been developed with the aim of enhancing immunogenicity, particularly cell-mediated and mucosal immunity. Although studies suggest that intradermal influenza vaccination may enhance immunogenicity compared to standard intramuscular vaccines in persons over 65 years of age (94), pooled analysis of RCT found no significant differences in seroprotection and seroconversion rates in older adults with intradermal vaccine compared to intramuscular (95) and intradermal influenza vaccines are no longer recommended. T cell responses were also not different between intramuscular versus intradermal injection in a randomized study comparing influenza vaccines in adults ≥ 65 years old (90).

It should be noted that when comparing different types of influenza vaccines, the formulation may differ. Current licensed inactivated influenza vaccines are manufactured using either split-virus or subunit formulations. They are all designed and licensed based on hemagglutinin antibody responses but while they may induce similar antibody responses, the differences become more evident when measuring cellular immune responses to vaccination (96). Split-virus vaccine lack some of the purification steps of subunit vaccines and therefore may contain a larger amount of internal viral proteins such as matrix protein (M1) and nucleoprotein (97) that are important to elicit T cell responses (98). Co et al. showed that the presence of influenza internal proteins, M1 and NP, contained in standard-dose split-virus vaccines but not in subunit vaccines, were necessary for stimulating CD8^+^ T cell responses measured by IFN-gamma production and by cytotoxicity assays *in vitro* (96). Importantly, a study evaluating the clinical effectiveness of split-virion versus subunit trivalent influenza vaccines in older adults using a case-positive, control test–negative study design, demonstrated a vaccine effectiveness of 77.8% (95% confidence interval [CI], 58.5%–90.3%) for the split-virion compared with 44.2% (95% CI, −11.8% to 70.9%) for the subunit vaccine (99). Unfortunately, there are not many studies performing head-to-head comparisons between the different available influenza vaccine options for older adults comparing both humoral and T cell responses. A randomized clinical trial comparing immunogenicity of currently available vaccine options for older adults—standard-dose quadrivalent vaccine, MF59-adjuvanted trivalent vaccine, high-dose trivalent vaccine, or recombinant-hemagglutinin quadrivalent vaccine – is currently under way and it will be important for identifying improved vaccination strategies for influenza in older adults (100).

### Herpes Zoster Vaccines

Herpes zoster results from the reactivation of latent varicella-zoster virus (VZV) infection. Although the reactivation of VZV can occur throughout life, the risk increases substantially with age and in conditions associated with a decline in T cell immunity. A live-attenuated VZV vaccine (Zostavax®) is approved for older adults to boost VZV-specific cell-mediated immunity (CMI). Evidence that the vaccine is partially effective in older patients comes from the Shingles Prevention Study that demonstrated a reduction in the incidence of herpes zoster and post-herpetic neuralgia by 51% and 67%, respectively (101). However, the efficacy of the vaccine was age-dependent, dropping from 64% in the age group 60–69 years to 41% in the age group 70–79 years. In addition to this, data on long-term follow-up indicates that vaccine-induced immune responses decline over time. Revaccination can have a booster effect although current evidence is not sufficient to support revaccination of older people (102).

A new adjuvanted recombinant zoster vaccine (Shingrix®) has been recently approved to prevent herpes zoster in older adults. It consists of recombinant VZV glycoprotein E and a liposome‐based AS01B adjuvant system. This system consists of two adjuvants, 3-O-desacyl-40-monophosphoryl lipid A (MPL) and QS-21 formulated in a liposomal delivery system (AS01B) (103). MPL is a TLR agonist, activating the innate immune system at the site of the injection and enhancing antigen-presentation (104). Whist the molecular mechanisms underlying the adjuvant effect of QS-21 are not yet fully understood, it has been demonstrated that it induces strong and persistent Th2 humoral and Th1 cell-mediated immune responses (105). It is thought that the use of liposomal formulations facilitates the escape of the antigen into the cytosol enhancing antigen-presentation through MHC-I pathway leading to cross-presentation to CD8^+^ T cells and an early IFN-gamma response that promotes vaccine immunogenicity (106). Interestingly, the AS01B adjuvant system seems to require the synergistic action of the three components together for optimal adjuvant effect (107). The efficacy of the adjuvanted recombinant vaccine has been demonstrated in two randomized placebo-controlled Phase III clinical trials, where the administration of two doses resulted in 97.2% protection against HZ in persons over 50 years of age (108) and 89.8% in adults over 70 years of age (109). While long-term follow-up is still ongoing, robust antibody and CD4^+^ T cell responses were found for at least 3 years after the vaccination, although CD8^+^ T cell correlates of protection were not identified (110). A meta-analysis comparing the two vaccines in adults over 50 years of age confirmed the superiority of the adjuvant recombinant subunit vaccine compared to the live attenuated vaccine for the prevention of herpes zoster infection despite a greater risk of adverse events at injection sites (111). An additional advantage of the recombinant zoster vaccine over the live-attenuated vaccine is its suitability to use in immunocompromised patients, including HIV-infected patients (112) and in transplant recipients (113).

### Pneumococcal Vaccines

The currently available 23-valent polysaccharide vaccine (PPV-23) has been used for many years in older adults and is still the first choice in many countries. However this vaccine does not generate adequate immunological memory, as purified polysaccharides do not induce persistent antigen-specific memory B cells (114). Furthermore, responses to PPV-23 were impaired in older adults compared to young individuals (115). A 13-valent conjugate vaccine (PCV-13) has been introduced and is now the first line choice for older adults in several countries as it has improved immunogenicity compared to the polysaccharide vaccine (116). Conjugation of polysaccharide antigens enables the uptake and antigen presentation in the context of MHC-II to CD4^+^ T helper cells resulting in the generation of memory B cells specific for the polysaccharides (114). A large randomized placebo-controlled trial demonstrated that the conjugate vaccine is effective in persons over 65 years of age, reducing the number of hospitalizations due to community-acquired pneumonia caused by vaccine-type strains by 45.6% and the number of cases of invasive pneumococcal disease by 75% (117). It is still debatable which pneumonoccal vaccine is more suitable to the elderly and this is largely reflected in the heterogeneity of the recommendations for pneumococcal vaccines from country to country. PCV-13 induces stronger and long-lasting memory responses compared to PPV-23, however PPV- 23 covers more serotypes. This is particularly relevant in the context of the serotype replacement that is seen as a consequence of routine childhood vaccination with PCV-13 leading to the reduction in the incidence of pneumococcal disease caused by vaccine serotypes while other serotypes become more prevalent (118).

### Tetanus and Diphtheria Vaccines

Antibody responses to tetanus and diphtheria vaccines are also suboptimal in old age. In addition to reduced antibody concentrations in the elderly, protection is short-lasting and a second booster after 5 years did not lead to additional long lasting immunity in older people (119).

Overall, immune responses to currently recommended vaccines are suboptimal in older people. Despite the important successes achieved with strategies currently in place to improve vaccine responses in the elderly, most available vaccines still fail to elicit long-lasting immune responses and insufficiently trigger cell-mediated and mucosal immunity. Therefore, novel approaches should be explored to enhance immunogenicity and efficacy of vaccines in this population.

## Novel Strategies for Enhancing Vaccine Responses

### Implementing New Correlates of Vaccine Efficacy

Although real estimates of vaccine efficacy can only be established in randomized, placebo-controlled trials against laboratory-confirmed cases, the standard of practice is to use surrogate markers of vaccine-induced protection against disease (120–122). Hemaglutinin inhibition (HI) assays detecting antibody responses to vaccine strains are the most widely used correlates of protection induced by vaccines. Nevertheless, studies in older adults have found a poor correlation between antibody responses to influenza vaccine and protection against laboratory-confirmed cases of influenza (7, 123). The limitations associated with over-reliance on HI assays to ascertain vaccine responses have been reviewed elsewhere (124), however there is growing appreciation that the use of HI antibody titers as a sole measure of vaccine efficacy may fail to detect important changes in cellular immunity that occur with age (6, 7). It has been shown that older adults exhibit lower T cell responses to influenza compared to young controls (125) and that preexisting CD4^+^ T cells against conserved internal influenza proteins are important for limiting virus replication and disease severity (126). Additionally, Sridhar et al. showed that, in the absence of crossreactive neutralizing antibodies, CD8^+^ T cells specific to conserved viral epitopes correlated with crossprotection against symptomatic influenza (127). However, T cell correlates of protection based on the frequency of IFN-gamma-producing CD4^+^T (126) and CD8^+^ T cells (128) have only been established in young adults and have not yet been validated in older adults. On the other hand, other studies have demonstrated that *ex vivo* T cell parameters (e.g., interferon (IFN)-gamma and IL-10 ratio, granzyme B levels) measuring cellular immune responses to influenza challenge performed better than antibody titers as correlates of vaccine efficacy in older adults (7, 129). Correlates of protection based on functional assays of CD8+ T cell cytolytic activity are important to better predict vaccine efficacy and should ideally be incorporated into the evaluation of protective immunity in the elderly (7). Nevertheless, there is still limited data on functional T cell responses to vaccines, particularly in older adults, such as CD8^+^ T cell-mediated *ex vivo* virus inhibition assays as described in HIV vaccine development (130). Although recent data indicates that innate immune cells may be important contributors for developing effective cytolytic-mediated immunity to infection this requires a functional readout of the response to vaccination.

Novel correlates of vaccine effectiveness are needed and an evolving area of interest is the contribution of neutralizing and cross-reactive antibodies induced by vaccination to enhanced protection against disease (131). The use of functional assays such as antibody-dependent cell mediated cytotoxicity (ADCC) and serum neutralization assays to detect cross-reactive antibodies that may not necessarily be detected in HI assays has been suggested as alternative correlates of protection however they are difficult to standardize across laboratories. Likewise, the incorporation of methods to assess antibody binding affinity, specificity, and epitope diversity of polyclonal antibodies would be important for a more comprehensive assessment of the quality of immunization-induced antibody responses and for developing more effective vaccines (132). Sequencing B and T cell receptors to analyze repertoire clonality and diversity could represent a valuable tool to predict vaccine efficacy by identifying vaccine-induced clones that will respond better and for longer to a given immunogen (133, 134). Although difficult to implement as routine measure of vaccine efficacy, assessment of repertoire clonality and diversity would be important to direct the development of next-generation vaccines that provide long-lasting immunity against infection.

### Searching for Novel Adjuvants to Stimulate the Immune System

Adjuvants act as enhancers of vaccine-induced immunogenicity at multiple dimensions: inducing local proinflammatory cytokine production, recruiting and activating innate immune cells, stimulating antigen presentation and ultimately boosting humoral and cellular immune responses (135). For many years, aluminium salts have been the only adjuvant in use in human vaccines. In recent years, high-throughput screening approaches have led to the discovery of many novel adjuvants. However, to date only two adjuvants (MF59 and AS01B) are currently licensed for persons older than 65 years, while the majority failed to translate to effective therapeutics mostly due to their side-effects (136). As our understanding of the mechanisms that boost immunogenicity rapidly increases, new adjuvants are being developed with focus on generating multifaceted immune responses. Recent research efforts have also focused on developing new ways to deliver old adjuvants in order to improve their function while reducing side-effects (137). The requirements for effective novel adjuvants are to boost innate and adaptive immune responses to vaccines and induce long-term protective memory as well as to counterbalance the low-grade inflammatory state that might hamper vaccine responses (136, 138). The incorporation of pathogens associated molecular patterns (PAMPs) in vaccine formulations that act as ligands for pattern recognition receptors (PRRs) on innate immune cells is a strategy already in place for enhancing vaccine-specific responses. PRR activation leads to inflammatory cytokine and type I IFNs production, facilitating antigen cross-presentation and activation of cytotoxic T cells (135). Due to their ability to induce strong cell-mediated responses, TLR ligands are attractive sources for developing new adjuvants (57, 139, 140). Some TLR agonists are already in clinical stage as vaccine adjuvants. Monophosphoryl lipid A is among the first of a new generation of TLR agonists to be already approved and in clinical use worldwide as an adjuvant in several vaccine formulations including a vaccine against hepatitis B virus (FENDrix) and human papilloma virus (Cervarix) (141). Another TLR4 agonist, glucopyranosyl lipid adjuvant (GLA) formulated in a squalene-in-water emulsion (SE), has been shown in a first-in-human trial to improve magnitude and quality of humoral and T-helper 1 type cellular responses elicited by the ID93 tuberculosis vaccine (142). The stimulatory effect of GLA-SE is well preserved in older adults (143) and *in vitro* studies in the context of vaccination with a split-virus influenza vaccine in older adults confirmed the activation of DCs to induce a Th1 response, increasing the interferon-γ to IL-10 ratio and the cytolytic (granzyme B) response to influenza virus challenge, both of which have been shown to correlate with protection against influenza in older adults (144). However, the response to TLR agonists was impaired in aged compared to young mice (145) and the age-related defects in TLR function and cytokine production might limit the utility of TLR ligands in older adults (58, 59). Although more research is needed, the use of combinations of TLR agonists has been proven effective in experimental models and might be a possible strategy for more effective vaccination in the older population (140).

### Triggering Innate Immune Memory

Effective vaccination strategies should aim at inducing protective adaptive immunity but also incorporate novel means of triggering innate immune memory to induce life-long protection against infection (146). Recent findings suggest that NK cells may play important roles in vaccination, through the modulation of adaptive immune responses and generation of innate immune memory (62, 63). NK cells can be activated following immunization through cytokines produced in response to adjuvants (147) or by direct stimulation of receptors, including TLRs (148). Thus, vaccine adjuvants can be optimized to promote activation and recruitment of NK cells to target tissues where they can positively or negatively regulate antigen presenting cells and downstream T cell responses (149). Additionally NK cells may contribute to enhanced vaccine responses through the generation of long-lived ‘memory’ NK cells capable of mediating rapid effector functions following re-exposure to antigen, reminiscent of T-cell memory responses (62, 150, 151). The concept of innate immune memory is relatively new and a better understanding of how memory NK cells are generated and can mediate specific recognition of antigen is important to define strategies promoting the development of these cells during vaccination.

### Targeting T Cells to Induce Broad Protective Immunity

An ongoing challenge in vaccination is the development of vaccines that are able to induce broad protective immunity. This is particularly relevant for influenza where next-generation vaccines inducing T cell immunity may potentially overcome the limitations of current available vaccines that rely on antibodies to provide narrow subtype-specific protection and are prone to antigenic mismatch with circulating strains. The concept of “universal” vaccines is based on the possibility of inducing heterosubtypic immunity, whereby T cells can target diverse influenza strains by recognizing highly conserved peptides (127, 152). Studies conducted during the 2009 H1N1 pandemic provided key insights into the role of cross-reactive T-cells in mediating heterosubtypic protection in humans. We conducted influenza studies to map T cell responses before and during infection in adults with no detectable antibodies to pandemic H1N1 and found that preexisting CD4^+^ T cells targeting highly conserved protein epitopes exhibited cytotoxic activity across strains and were important to limit viral replication and disease severity (126). By mapping the type of epitopes that were able to generate heterotypic responses across strains, the results of this work and others (153) can aid the development of broadly protective T cell vaccines (154). This may be particularly important in the context of pandemics where there is no preexisting immunity. Interestingly, a recent study done in COVID-19 convalescent patients detected circulating SARS-CoV-2-reactive CD4^+^ T cells in 40%–60% of unexposed individuals, supporting the importance of cross-reactive heterotypic T cell responses for clinical protection and limiting disease severity (155).

### Exploring New Pathways for the Development of Broadly Protective Vaccines

Innate T-cells (MAIT cells, γδ cells, and NKT cells) are attractive vaccine targets as they can link both innate and adaptive immunity by mediating TCR-dependent and independent (innate-like) functions (156). A common feature of innate T cells is their capacity to respond rapidly to danger signals and pro-inflammatory cytokines (such as IL‐12, ‐15, ‐18 and Type I IFNs) in a TCR‐independent mechanism and participate in the early stages of defense against certain infections. MAIT cells are abundant in human lungs where they have been shown to contribute to protection against influenza infections (157) and mucosal tissues, such as the intestinal mucosa, making them attractive targets for mucosal vaccine design. Recent studies have shown that MAIT cell frequencies can be rapidly ‘boosted’ through mucosal administration of synthetic MAIT cell ligands with TLR agonists (157, 158) and this could be particularly beneficial for the elderly who have impaired MAIT cell immunity.

Bystander activation by cytokines is a feature shared by a subset of conventional T cells, particularly CD8^+^ T cells. We have recently shown that as T cells differentiate toward senescence they become less responsive to TCR conventional signaling while acquiring innate-like functions (32). The reprogramming of highly differentiated CD8^+^ T cells from TCR to NKR functional activity provides them broad protective functions that can be beneficial in the context of aging (35) and might be also relevant for vaccination.

Another area of potential interest is the use of monoclonal antibodies that selectively block inhibitory receptors to boost T cell function. In light of the unprecedented results obtained with the use of checkpoint inhibitors (e.g., PD-1, CTLA-4) in cancer, new avenues of research are open for the use of these immunomodulators in other settings, including vaccination (159, 160). Interestingly, improved vaccine responsiveness has been linked to reduced frequencies of CD4^+^ and CD8^+^ T lymphocytes expressing PD-1. For instance, immunological responses to the live-attenuated zoster vaccine in individuals over 50 years of age were correlated with pre-vaccination levels of regulatory T cells and PD1-expressing T cells, regardless of the age of the vaccine (161). *Ex vivo* blocking experiments corroborated a role of PD1 and CTLA4 as modulators of decreased VZV responses (161). A study on the responses to a trivalent inactivated influenza vaccine in lung cancer patients receiving PD-1 blockade therapy compared to age-matched healthy controls showed comparable serological protection but an increased rates of immune-related adverse events (IRAEs) (162) although a subsequent study found no increase in incidence or severity of IRAEs in patients on immune checkpoint inhibitors who received the flu vaccine (163). While more research is needed on the safety and efficacy of such combinations of immune checkpoint inhibitors with vaccines, this combinatorial approach has been tested and proved efficient in preclinical and clinical trials using therapeutic cancer vaccines with anti-PD1 (164, 165) or anti-CTL4 (166) monoclonal antibodies. As the expression of inhibitory receptors on T cells has been shown to increase with age and differentiation (37, 167) the selective blockade of inhibitory receptors known to regulate T cell activity could be explored as means of boosting cellular responses in the elderly prior to or during vaccination.

### Blocking Baseline Inflammation to Boost Vaccine Responses

Responses to vaccination vary widely across individuals and are generally poorer in particular groups including not only the elderly but also individuals with autoimmune diseases, HIV infection (168) and cancer (169). A common feature among these groups is the presence of a chronic inflammatory background that has been associated with adverse health outcomes (170). Furthermore there is a growing appreciation that pre-existing inflammation may be a determinant of vaccine responsiveness and thus modulating baseline inflammation prior to vaccination has become an attractive area of research to boost vaccine responses (16, 83, 171). Using high-throughput technology researchers have identified baseline transcriptional signatures that predict protective immune responses to vaccines (76, 78–81). Most of the signatures identified so far are indicative of broad immune activation and excessive inflammation. For example, a study comparing responses to the yellow fever vaccine in an African cohort compared with a Swiss cohort found that an activated immune profile of NK cells, monocytes and differentiated T and B cell subsets was associated with reduced responses to vaccination (81). Our group has previously shown that older individuals have decrease ability to mount recall responses to VZV antigen challenge in the skin (172) and this was subsequently associated with increased baseline local inflammation (79). Ingenuity pathway analysis indicated that this inflammation was driven by the activation of p38 MAP kinase pathway in the skin of old individuals compared with young. Short-term systemic treatment with an oral p38 MAPK inhibitor (Losmapimod) significantly increased the cutaneous VZV response in older subjects (79), supporting the concept that anti-inflammatory interventions may be promising strategies for boosting immunity during aging. Furthermore, oral administration of an mTOR inhibitor (Rapamycin) prior to influenza vaccination of older adults resulted in increased antibody titers against all three strains of a trivalent influenza vaccine by more than 20% in individuals aged above 65 years (173). Other immunomodulator agents such as metformin, imiquimod (174) and anti-inflammatory drugs inhibiting COX2 expression (175) (e.g., aspirin and NSAIDS) that are currently approved for clinical use in other settings may represent attractive approaches to promote more effective vaccine responses by transiently alleviating chronic inflammation prior to vaccination. Finally, it is likely that targeting other sources of inflammaging by changing the composition of the microbiome (176) or selectively removing senescent cells using senolytic drugs (177) may represent further opportunities for enhancing vaccine immunity in the setting of chronic inflammation.

## Reflections on COVID-19 Vaccination Strategies for the Elderly

The discussion about the impact of aging on immunity and vaccination is particularly relevant at the moment as the COVID-19 pandemic placed again the spotlight on the vulnerability of older adults to emerging infectious diseases. Epidemiological data reveals that individuals over 60 years of age are disproportionately affected by SARS-CoV-2 infection experiencing the most severe forms of disease and the highest hospitalization rates (178–180). Age is a strong predictor of death among patients hospitalized with COVID-19 (181, 182) and a review of epidemiological data from different countries revealed an exponential increase in case fatality rates with age, regardless of the geographic region (183). Despite being the most affected risk group, older adults are the least likely to respond to a new vaccine. This represents a major challenge for vaccine development and thus it is critical to understand how immunosenescence and inflammaging impact on vaccine responses to ensure that vaccination remains effective in this age group (184). To meet this need, leading vaccine developers Oxford University/AstraZeneca (ClinicalTrails.gov number: NCT04516746), NIAID/Moderna Therapeutics (NCT04405076) and BioNTech/Pfizer (NCT04368728) are currently recruiting adults over 55 years of age to evaluate efficacy, safety and immunogenicity of their vaccine candidates in older individuals. However, due to intricacies of clinical trial design with strict inclusion/exclusion criteria most COVID-19 vaccine studies may fail to include a sufficient number of older individuals, in particular those in their 70s and 80s. As of 3 of September 2020, the COVID-19 vaccine development landscape includes 33 vaccine candidates in clinical trials, of which 6 candidates are currently in phase III clinical trials (185). Despite the promising preliminary reports of their phase I/II trials (186, 187), current vaccine front-runners have not yet published results on the vaccine safety and immunogenicity in elderly. Relaxing the eligibility criteria and ensuring an adequate representation of the groups most affected by COVID-19 disease - such as elderly people, those with comorbidities and people from black, Asian and minority ethnic groups – is of key importance for successful vaccination strategies for COVID-19.

Trials in older adults are also important to understand why immune responses to COVID-19 infection and vaccination may vary from person to person. A recent study performed deep immune profiling of 125 COVID-19 patients and identified immune profiles associated with poor clinical outcomes (97). Severe COVID-19 disease was associated with an immunotype characterized by the paucity of circulating follicular helper cells and the presence of highly activated CD4^+^ and CD8^+^ T cells, with increased frequencies of highly differentiated CD8^+^ T cell “EMRAs” and exhausted PD1^+^ CD8^+^ T cells, providing evidence for the association between an immunosenescent phenotype and disease severity. Other studies have shown that severe COVID-19 disease correlated with elevated serum concentrations of inflammatory cytokines including interleukin-6 (IL-6), granulocyte colony-stimulating factor (G-CSF), IP-10, MCP1, macrophage inflammatory protein 1α (MIP1α) and tumor necrosis factor (TNF) (188–191). Among these, IL-6 has received particular attention (189) providing support for several clinical trials on IL-6 receptor antagonists as potential treatments for severe COVID-19 disease (192). Accumulating evidence suggests that the pathophysiological hallmark of COVID‐19 disease is severe inflammation with descriptions of a cytokine storm syndrome (193, 194) induced by a dysregulated monocyte/macrophage response (195, 196). As previously discussed, the presence of low-grade sterile inflammation characterized by high baseline serum concentrations of pro-inflammatory cytokines including IL-6 is a hallmark of aging (70) and is predictive of early mortality (73). Thus, it can be speculated that inflammaging is one of the mechanisms underlying increased morbidity and mortality due to SARS-CoV-2 infection in older adults (196). As pre-existing inflammation may also be detrimental to vaccine responses it has been proposed that reducing inflammation with short-term course of mTOR or p38 MAPK inhibitors and possibly other anti-inflammatory agents (e.g., steroidal drugs such as dexamethasone) may be used as a strategy for improving COVID-19 vaccine responses in older people (84).

## Concluding Remarks and Unsolved Questions

Despite the important successes achieved with current vaccines, most available vaccines still fail to elicit long-lasting immunity in older adults. Current vaccine strategies must evolve to be able to enhance cell-mediated and mucosal immunity in addition to inducing long-lasting antibody responses. However, to date most clinical trials leading to vaccine approval in older adults rely entirely on antibody responses as correlates of protection and thus novel correlates of vaccine effectiveness are needed that fully reflect the changes occurring with age in the immune system. The use of system vaccinology approaches can aid researchers in identifying signatures that predict protective immune responses and this information can be used for optimization of current vaccination strategies. Responses to vaccination vary widely across individuals and baseline immune profiles matter to determine the outcome of vaccination. Recent data suggests that excessive baseline inflammation is deleterious and may hamper immune responses and thus novel approaches aimed at reducing inflammation may offer novel opportunities to improve vaccine responses in older individuals. Yet the prevailing view is that adjuvants improve vaccine responses by promoting local inflammation. Thus more research is needed to understand the role of inflammation in vaccine responses and to reconcile these seemingly paradoxical observations. It could be speculated that the effects of systemic versus local inflammation are distinct and that the beneficial effects of anti-inflammatory drugs on vaccine response result from the systemic reduction of the low-level chronic inflammation. Additionally, chronic immune activation may be associated with desensitization or tolerance to new antigenic stimulation resulting in poor immune responses. Thus stronger adjuvants may be needed to overcome this tolerogenic state and alleviate the consequences of chronic inflammation. There is a need to develop newer and more specific adjuvants, able to fine tune immune responses and selectively stimulate pathways that lead to long-lasting immune protection. As our understating of immunosenescence and inflammaging increases new individualized approaches could point towards the development of more effective vaccines for older individuals.

## Author Contributions

BP has done the literature search and writing. AA and X-NX contributed for the writing and revision of the manuscript. All authors contributed to the article and approved the submitted version.

## Funding

BP is funded by the Joint Research Committee of Chelsea and Westminster Hospital NHS Foundation Trust and Imperial College London, UK. AA was supported by the Medical Research Council (MRC) Grand Challenge in Experimental Medicine (MICA) Grant (MR/M003833/1). X-NX was supported by the Department of Health and Social Care using UK Aid funding and is managed by the Engineering and Physical Sciences Research Council (EPSRC, grant number: EP/R013764/1); Note: the views expressed in this publication are those of the author(s) and not necessarily those of the Department of Health and Social Care).

## Conflict of Interest

The authors declare that the research was conducted in the absence of any commercial or financial relationships that could be construed as a potential conflict of interest.
